# Influence of PVA Molecular Weight and Concentration on Electrospinnability of Birch Bark Extract-Loaded Nanofibrous Scaffolds Intended for Enhanced Wound Healing

**DOI:** 10.3390/molecules25204799

**Published:** 2020-10-19

**Authors:** Francis Kamau Mwiiri, Rolf Daniels

**Affiliations:** Department of Pharmaceutical Technology, Eberhard Karls University, Auf der Morgenstelle 8, 72076 Tuebingen, Germany; f.kamau@gmx.de

**Keywords:** electrospinning, PVA, molecular weight, birch bark extract, wound dressing

## Abstract

Triterpenes from the outer bark of birch (TE) are known for various pharmacological effects including enhanced wound healing. Apart from an already authorized oleogel, electrospun nanofiber mats containing these triterpenes in a polyvinyl alcohol (PVA) matrix appear to be an advantageous application form. The effects of PVA molecular weight and concentration on the fiber morphology have been investigated. Three different molecular weights of PVA ranging from 67 to 186 kDa were used. The concentration of PVA was varied from 5 to 20 wt%. Polymer solutions were blended with colloidal dispersions of birch bark extract at a weight ratio of 60:40 (wt.%). The estimated viscosity of polymer solutions was directly linked to their concentration and molecular weight. In addition, both pure and blended solutions showed viscoelastic properties with a dominant viscous response in the bulk. Fiber morphology was confirmed using scanning electron microscopy (SEM). Both polymer concentration and molecular weight were found to be significant factors affecting the diameter of the fibers. Fiber diameter increased with a higher molecular weight and polymer concentration as more uniform fibers were obtained using PVA of higher molecular weight (146–186 kDa). In vitro drug release and ex vivo permeation studies indicated a faster drug release of betulin from electrospun scaffolds with lower PVA molecular weight. Our research suggests that the fabricated TE-loaded PVA electrospun dressings represent potential delivery systems of TE for wound care applications.

## 1. Introduction

Once the skin is damaged, very important skin functions such as physical protection and thermoregulation are lost which can result in either acute or chronic wounds. Most of the wound dressings in the market have their basic functions to provide a protective barrier against bacterial contamination and absorb exudate [1]. Thus, developing wound dressings for not only covering wounds but with loaded active ingredients to improve the wound healing process would be greatly beneficial. The pentacyclic triterpenes from outer birch bark extract are known for their pharmacological effects such as anti-inflammatory, antimicrobial, anti-viral, anti-cancer activity and wound healing effects [2,3,4,5,6,7,8,9]. A suitable triterpene dry extract (TE) from the outer bark of birch contains >80% (*w*/*w*) betulin as the main component and is obtained by accelerated solvent extraction with n-heptane [10]. Other disclosed triterpenes of this dry extract include lupeol (LU), erythrodiol (ER), betulinic acid (BA) and oleanolic acid (OA) (Table 1) [11]. However, TE exhibits low solubility in polar and non-polar solvents that may lead to a poor bioavailability which might limit its therapeutic application [8,12]. Currently, topical formulations containing TE include water-in-oil foams [9], cosmetic water-in-oil creams (Imlan Creme pur, Birken AG) and a TE oleogel based on sunflower oil received from the European marketing authorization in January 2016 [13]. Notably, TE together with sunflower oil supports more wound healing than in combination with other oils as examined by Steinbrenner et al. [8]. The efficacy of TE-based formulations has been well investigated in vivo on different types of wounds including dystrophic epidermolysis bullosa, where treatments promoted wound healing [14,15,16]. A study conducted by Ebeling et al. showed the molecular mechanism of the effects of birch bark extract on wound healing properties in human primary keratinocytes and porcine ex vivo wound healing models. They showed that TE and betulin mainly accelerated re-epithelialization by enhancing migration of keratinocytes and their differentiation. Beyond that, they found that TE led to upregulation of proinflammatory mediators such as COX-2 and IL-6 which play a key role in wound healing and epidermal barrier repair [6]. In addition, TE positive effects have been observed in all three phases of wound healing [13].

By use of the electrospinning technique, it is possible to prepare fibers with a diameter from a few micrometers down to several nanometers [17,18]. These nanofibers have interesting characteristics, e.g., high surface area to volume ratio, and can form mats/fleeces with high porosity which makes them attractive materials for wound dressing [19]. Additionally, electrospun nanofibrous scaffolds tend to mimic the structure of the native extracellular matrix (ECM), hence they facilitate cell proliferation, improve gaseous exchange, assist in the removal of exudate and act as a physical barrier against entry of microorganisms during wound healing with eventual tissue repair of damaged tissues [19,20,21,22,23].

PVA is a water-soluble, non-toxic, biocompatible and biodegradable hydrophilic polymer with good chemical and mechanical properties, and has been approved by the U.S. Food and Drug Administration (FDA) for both biomedical and pharmaceutical applications [24,25]. Many researchers have investigated the influence of various parameters on fiber morphology and diameters of electrospun PVA fibers. Some of these parameters include polymer molecular weight and solution concentration [26,27]. PVA polymer has either been used alone or combined with other biodegradable polymers and electrospun to form scaffolds for various applications. Furthermore, PVA has also been blended and electrospun with active ingredients such as hyaluronic acid/L-arginine, soursop leaves extract, silver nanoparticles and curcumin for wound healing [28,29,30,31]. Recently, we have developed, for the first time, a PVA bioactive wound dressing containing TE which showed accelerated wound healing [32]. In this present work, we investigated the influence of PVA molecular weight on TE-loaded electrospun nanofibers.

The purpose of this work was to (1) assess the effect of PVA molecular weight, blend composition and solution concentrations on rheological properties and the resulting fiber morphology, and (2) investigate the relationship of rheological properties and electrospinnability of polymer solutions. Rotational and amplitude sweep tests of the prepared solutions were performed. The surface properties of electrospun fiber mats were characterized by SEM. We also conducted ex vivo permeation and in vitro release studies using Franz diffusion cells to test the biopharmaceutical characteristics of betulin containing electrospun nanofiber mats.

## 2. Results and Discussion

The electrospinnability of polymer solutions is known to depend strongly on their viscosity, surface tension and electrical conductivity. This might be affected further by the properties of an active compound which is added to the matrix forming polymer. In this study, the active compound is added as a colloidal dispersion consisting of TE, Phospholipon 90H (PL90H) and sunflower oil (SO) dispersed in water [33]. Therefore, the properties of PVA solutions and blended dispersions were assessed and related to their ability to form electrospun fibers.

### 2.1. Rheological Characterization of the Electrospinning Solutions

**Figure 1:**
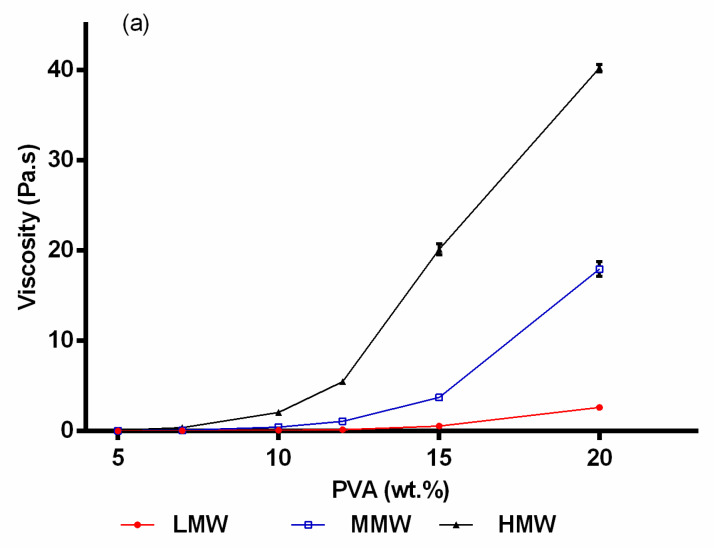

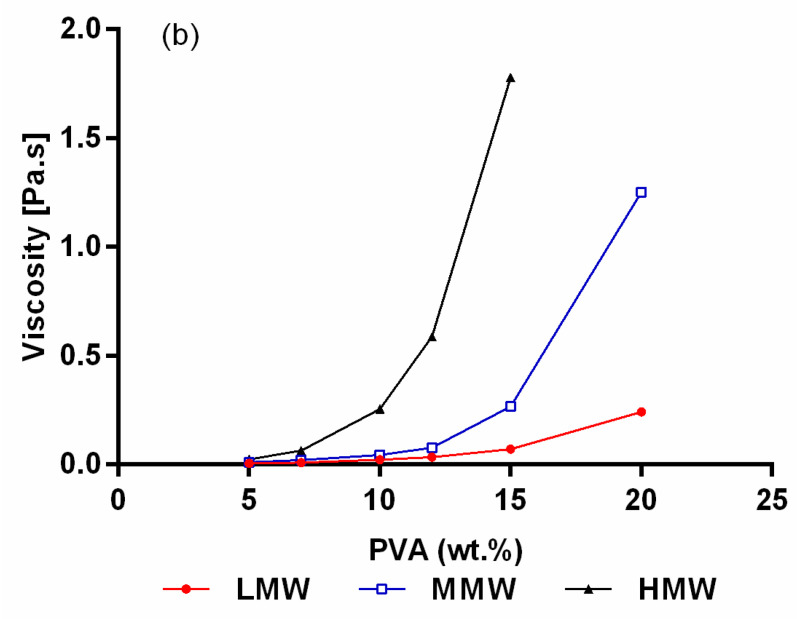
(**a**) Influence of molecular weight and PVA concentration on viscosity. (**b**) Viscosity of blended PVA/TE dispersions solutions in the ratio of 60:40 (error bars are smaller than the symbol size).

**Figure 2:**
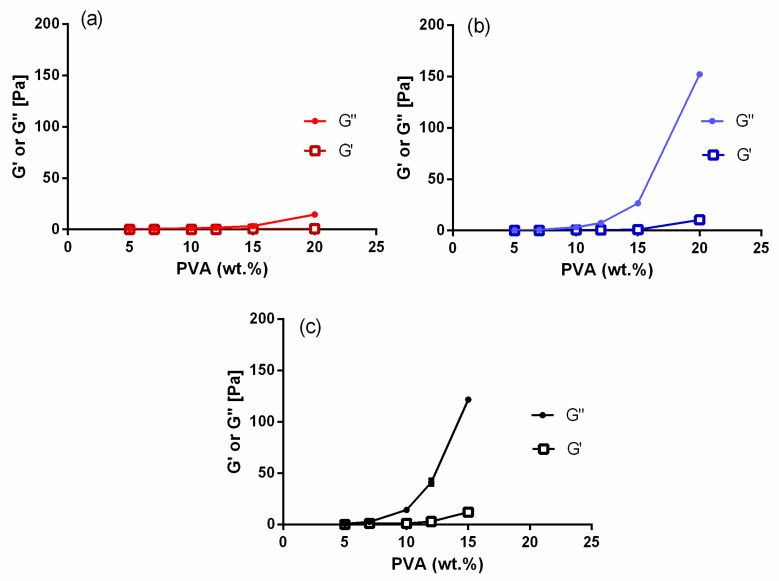
Storage (G′) and loss (G″) moduli as a function of PVA concentration (wt.%). (**a**) Low molecular weight (LMW), (**b**) medium molecular weight (MMW) and (**c**) high molecular weight (HMW) (error bars are smaller than the symbol size).

Rotational and amplitude sweep tests were performed on all PVA/TE blended solutions as well as on plain polymer solutions. Figure 1a shows the results obtained from rotational tests, where changes in viscosity as a function of concentrations for pure polymer solutions with different molecular weights are presented. As expected, solution viscosity clearly increases significantly (*p* < 0.05) with increasing polymer concentration and molecular weight. For example, the viscosity of the pure PVA solutions (5–20 wt.%) varied from 0.012 to 2.43 Pa × s (low molecular weight (LMW)), 0.035 to 17.9 Pa × s (medium molecular weight (MMW)) and 0.09 to 40.2 Pa × s (high molecular weight (HMW)). The results presented in Figure 2 show the viscoelastic properties of the pure polymer solutions (5–20 wt.%) with their molecular weights. It can be observed that the loss modulus (G″) and storage modulus (G′) increase consistently with an increase in PVA concentration and molecular weight. Additionally, solutions with low PVA concentrations (<10 wt.%) showed a steep decline in their storage modulus (G′) already at weak deformation. Notably, G″ dominates always over G′, indicating that PVA forms predominantly viscous liquids. Similar observations were also made elsewhere [26,34,35]. 

**Figure 3:**
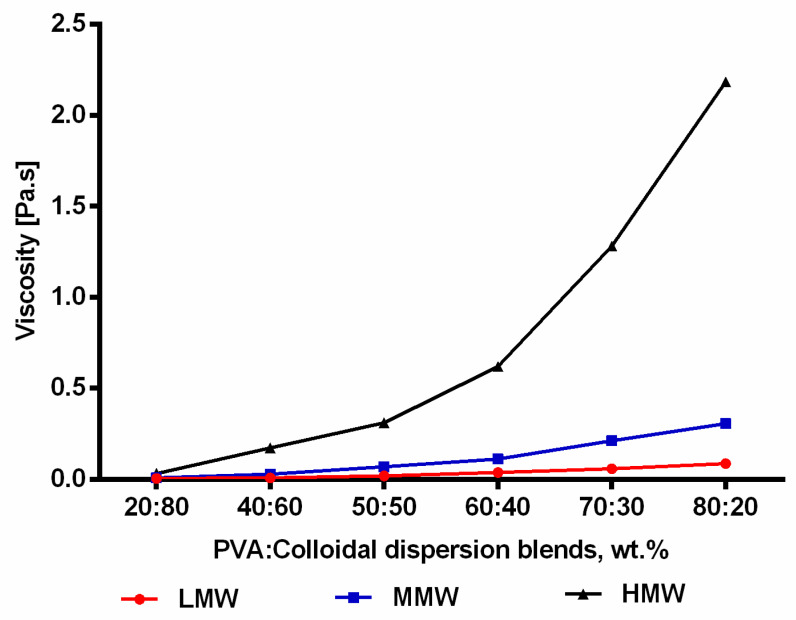
Viscosity as a function of PVA/colloidal dispersion aqueous blends (error bars are smaller than the symbol size).

As expected, blending of pure polymer solutions with colloidal dispersions (60:40) resulted in a reduction in the viscosity (Figure 1b). The viscosity is almost the same as for plain PVA solutions with the respective lower concentration indicating that there is no specific interaction of PVA and the colloidal dispersion. A similar reduction in viscosity has been reported after blending PVA with other agents [36,37,38]. Figure 3 shows the results from rotational tests when 12 wt.% of all molecular weights was blended with the colloidal dispersion in different compositions (20:80, 40:60, 50:50, 60:40, 70:30, 80:20). It can be clearly seen that the viscosity increased significantly (*p* < 0.05) as the amount of PVA increased in the blend. Again, there are no hints that the colloidal TE dispersions interact specifically with PVA. PVA/TE dispersion blended solutions maintained their viscoelastic characteristic, with the loss modulus (G″) always being greater than the storage modulus (G′). Further, both G″ and G′ values increase as the molecular weight increases in the blend composition. 

Not surprisingly, polymer concentration, molecular weight and viscosity are correlated with each other. An increase in polymer solution concentration will result in greater polymer chain entanglement since polymer chain length or molecular weight is directly proportional to the number of entanglements [26,27,39].

### 2.2. Surface Tension and Conductivity of Polymer Solutions

**Figure 4:**
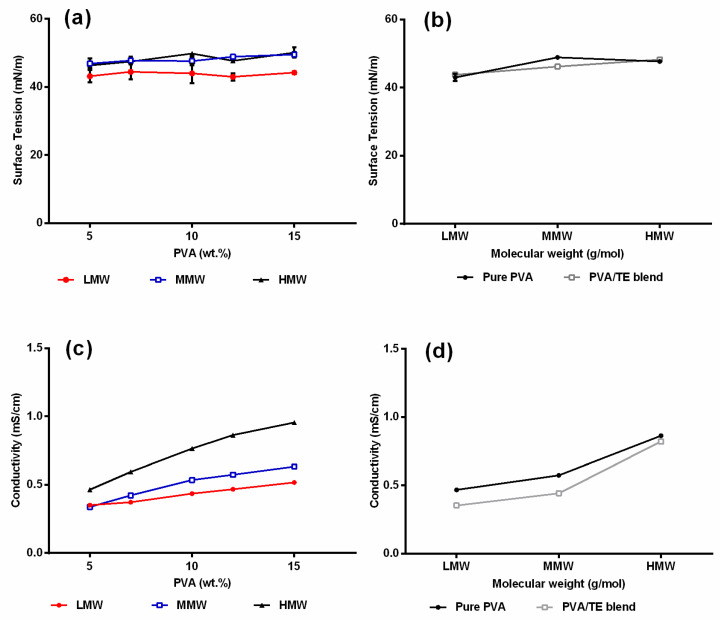
(**a**) Surface tension and (**c**) conductivity as a function of PVA concentration and molecular weight. (**b**) Surface tension and (**d**) conductivity of blended PVA/TE solution (60:40) at different molecular weights (error bars are smaller than the symbol size).

Electrospinning only occurs when the charges applied on the polymer solution are high enough to overcome the surface tension. Therefore, a low surface tension is favorable in electrospinning as this minimizes the amount of critical voltage needed for the jet formation from the Taylor cone. The results in Figure 4a clearly show that the used PVA solutions are surface-active being related to H-bonding between hydroxyl groups on PVA chains and water [40]. The surface tension of the polymer solutions remained nearly unchanged regardless of the polymer concentration and molecular weight used, indicating that the surface is completely saturated with the polymer in the whole relevant concentration range. The conductivity values in Figure 4c increased gradually as the amount of PVA concentration and molecular weight increased. This can be attributed to the fact that sodium acetate is a major impurity during production of PVA, dissociating into sodium and acetate ions in water, and therefore conductivity increases as a higher amount of PVA is added [41,42]. As expected, the addition of the colloidal TE dispersion which comprised only substances which are practically insoluble in water did neither affect the surface tension nor the electrical conductivity (Figure 4b,d), the latter being only reduced according to the reduced fraction of PVA in the blend. With respect to electrospinnability, it is known that both a too low or a conductivity beyond a critical value, for example, through addition of salts, e.g., NaCI, will hinder Taylor cone formation and the electrospinning process [43,44]. The measured values (0.34–0.96 mS/cm) are within a range that are reported to be suitable for electrospinning [45,46].

### 2.3. Qualitative Analysis of Electrospinning Process and Nanofiber Formation

Pure polymer solutions at lower concentrations < 10 wt.% of LMW showed unstable Taylor cone and discontinuous jet during electrospinning. Only at 12 wt.%, a stable jet was observed. Beyond this concentration, Taylor cone and jet instability occurs. The same phenomena were observed with MMW, where below 7 wt.% PVA polymer solution, an unstable jet and Taylor cone were formed. Between 10 and 12 wt.%, the jet stability improved. Beyond 15 wt.%, unstable jet formation can be observed. Pure polymer solutions of HMW between 5 and 7 wt.% exhibited stable continuous jet, whereas they destabilized between 10 and 15 wt.% and became discontinuous. After a prolonged electrospinning process with polymer solutions of high concentrations, the Taylor cone dries out and clogging of the needle tip was observed. With the polymer solution at 20 wt.% of HMW, the solution did not flow through the needle tip, while jet formation only occurred for a few seconds and the electrospinning process stopped. No jet formation with PVA/TE blends of LMW at low concentrations (<12 wt.%), <10 wt.% of MMW and <7 wt.% of HMW was observed. Blends of 20 wt.% in LMW, 12–15 wt.% in MMW and 10–12 wt.% in HMW had good performance in the formation of a continuous jet during the electrospinning process even after a long duration (4 h). 

**Figure 5:**
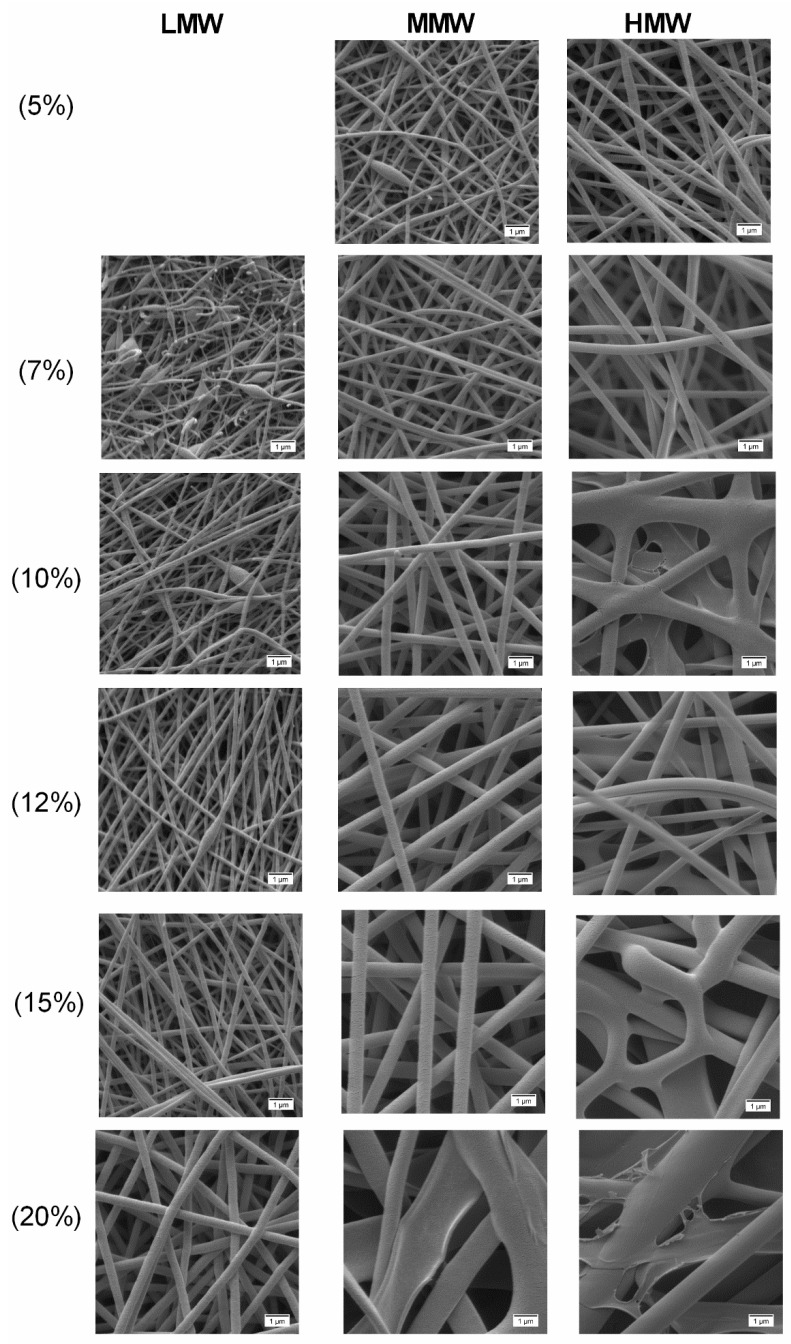
SEM images at 10,000× showing the obtained electrospun fibers from pure PVA polymer solutions. All scale bars represent 1 µm.

**Figure 6:**
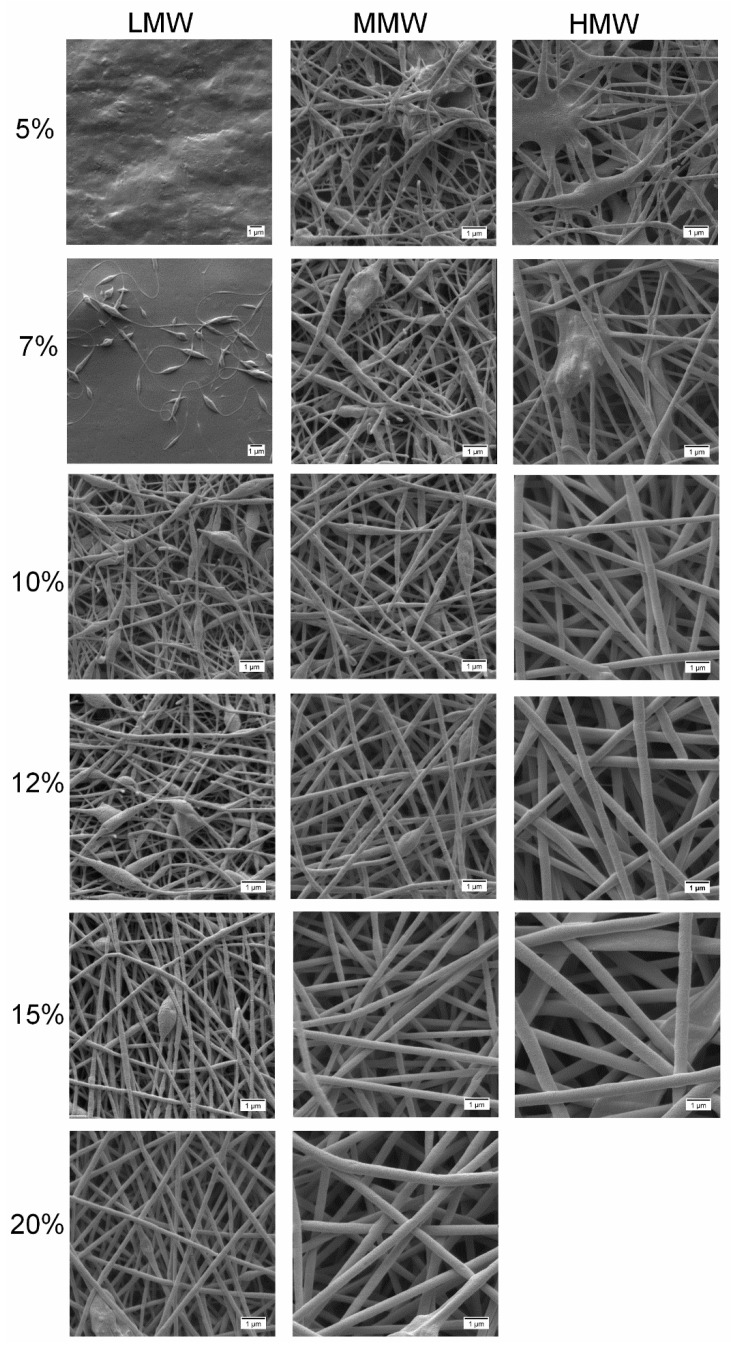
SEM morphologies at 10,000× of loaded electrospun PVA/TE blend (60:40) fibers at different concentrations and molecular weights. All scale bars represent 1 µm.

The success of the electrospinning process is strongly correlated with the characteristics of the polymer solutions from which the fibers were produced. Hence, with the change in molecular weight and concentration, a difference in the electrospinnability and morphology of the obtained fibers was expected. For instance, in the case of pure PVA solutions (Figure 5), bead-free fibers were obtained with solutions at lower concentrations (5–7 wt.%) of HMW and 7–15 wt.% for MMW, while for LMW in the range of 12–20 wt.%. While electrospinning pure polymer solutions at high concentrations of 10–20 wt.%, particularly of HMW, the high viscosity prohibits the continuous flow of the polymer solution jet towards the collector. Even practical handling of such a kind of solution was difficult. At 5–7 wt.% of HMW blended solutions (Figure 6), due to low viscoelasticity, only beaded fibers were formed. Once the PVA solution concentration was increased to 10–15 wt.%, the bead defect was completely removed and smooth fibers were formed. The solutions with low viscosity (<0.09 Pa × s), mainly those of LMW or low amounts of PVA in blend composition, produced a mixture of fibers and beads. Only droplets were produced instead of jet formation, due to the jet breaking up into droplets similar to electrospraying. This indicated that by increasing the polymer concentration of the solution, chain entanglement increases among polymer chains which overcome the surface tension and bead-free fibers can be produced [26,43,47]. We hereby conclude that polymer solutions or blends should show greater viscous properties over elastic behavior in the bulk, but only a proper relation between these two characteristics enables jet and fiber formation during the electrospinning process.

### 2.4. Effect of Concentration and Molecular Weight on Fiber Morphology

Figure 5 shows electrospun fibers produced from pure PVA polymer solutions. SEM analysis showed drastic changes in terms of fiber morphology when the molecular weight and concentration of polymer solutions were changed. In LMW, no fibers were formed over the collector with the polymer solution of 5 wt.% due to the low viscosity. With higher concentrations of 7 wt.% and 10 wt.%, a mixture of fibers with beads was formed. From 12 wt.% PVA, uniformity of fibers increased, where at 20 wt.% PVA, smooth uniform thicker fibers were observed instead of beaded fibers. For MMW, the formation of beaded fibers was only observed at 5 wt.% PVA. With 7 to 15 wt.% polymer solutions, smooth, uniform and bead-free fibers were produced. Flat and thicker fibers were observed with the polymer solution of 20 wt.% due to its high concentration.

In the case of HMW, a different pattern was observed, where only between 5 and 7 wt.% PVA, smooth, uniform fibers were produced. Besides that, between 10 and 20 wt.% PVA, fiber uniformity decreased and resulted in flat non-uniform thicker fibers with a few interconnected web-like structures in several points. Further, the observed poor fiber morphology of unloaded electrospun fibers (Figure 5) especially with HMW at high concentrations (10 to 20 wt.%) was due to the high viscosity which caused resistance to jet stretching during the electrospinning process. 

The measured fiber diameter indicated that the fiber diameter not only increased across polymer concentrations but also with the increase to the higher molecular weight. Pure electrospun fibers produced with LMW had average diameters between 147 (10 wt.%) and 390 nm (20 wt.%), MMW ranged between 183 (5 wt.%) and 1357 (20 wt.%) nm and HMW ranged between 269 (5 wt.%) and 2204 nm (20 wt.%) (Table 2). In accordance with Koski et al. [26], this finding shows that polymer concentration and molecular weight are influential in determining whether fibers or beads are produced. 

### 2.5. Incorporation of TE Colloidal Dispersions into Nanofibers 

Figure 6 shows the examined SEM morphologies after blending of differently concentrated pure PVA solutions with colloidal dispersions (60:40), where different structures with varied molecular weight and concentration were observed. The results clearly show that not only the morphology of electrospun fibers but also the resulting fiber diameter was changed after PVA was blended with the aqueous colloidal dispersions. As can be seen, with blends of LMW at 5 and 7 wt.%, no fibers were formed due to the low viscosity. By increasing the concentration from 10 to 20 wt.%, fiber uniformity increased gradually as the blend at 20 wt.% produced more uniform fibers. In the case of MMW, blends at 5 and 7 wt.% produced a mixture of fibers and beads, whereas at 10 to 12 wt.%, uniformity increased with the presence of a few beads. Blends at 15 and 20 wt.% produced smooth, uniform fibers without beads. 

Similar observations were made with blends of HMW at 5 to 7 wt.%, where a mixture of beads and fibers was formed. In contrary, smooth, uniform homogenous fibers were already achieved at 10 to 15 wt.%. Blends of electrospun fibers produced with LMW had average diameters between 143 (12 wt.%) and 241 nm (20 wt.%), MMW ranged between 174 (10 wt.%) and 424 nm (20 wt.%) and HMW ranged between 319 (10 wt.%) and 605 nm (15 wt.%) (Table 3). Overall, the addition of colloidal dispersions resulted in a decrease in the average fiber diameter to submicron range < 1 µm due to the viscosity reduction of polymeric solutions. Moreover, and as also reputed by other groups, the reduced viscosity contributed to better stretching of the jet and improved fiber morphology with smooth, thinner fibers [36,48,49]. Furthermore, formation of smooth, uniform fibers without beads indicated that colloidal dispersions were entrapped in the fibers and were within the range of the carrying capacity of the fibers [32].

### 2.6. Fiber Morphology in Different Compositions

**Figure 7:**
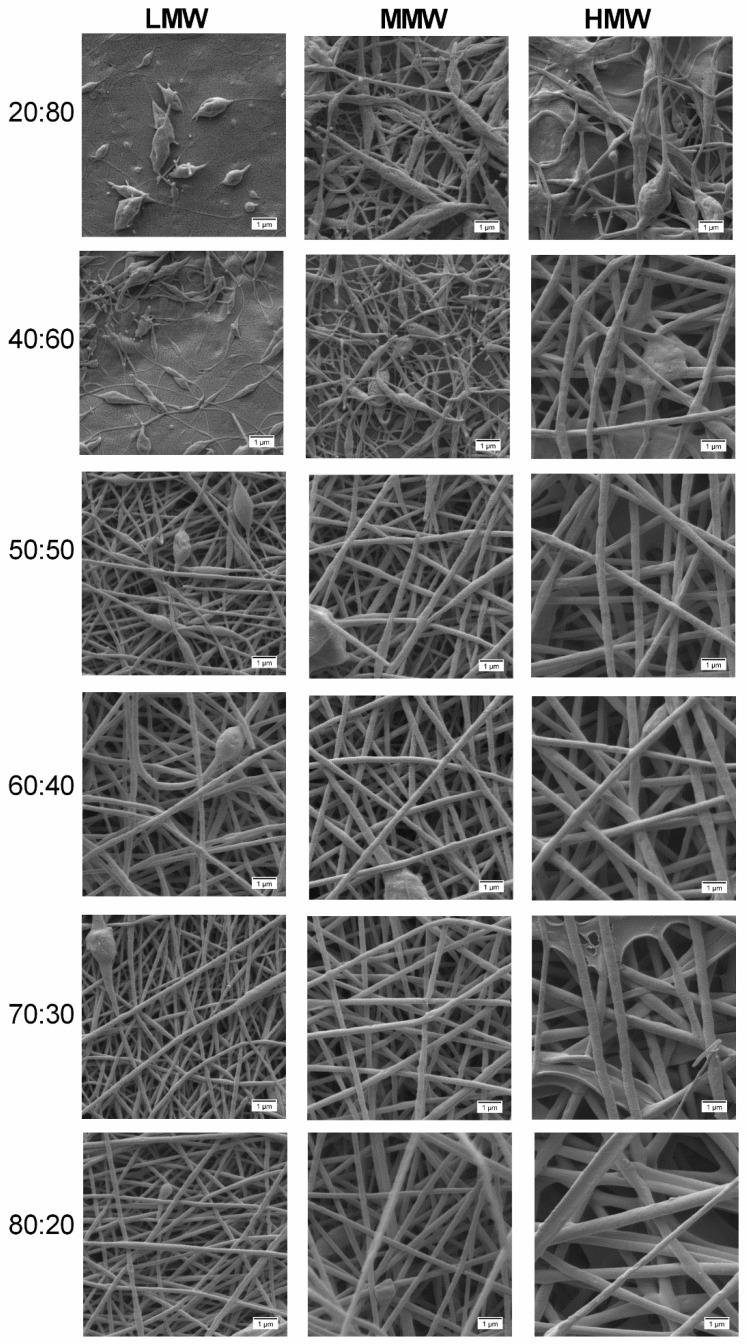
SEM morphologies at 10,000× of electrospun 12 wt.% PVA/colloidal dispersion fibers at different compositions and molecular weights. All scale bars represent 1 µm.

The polymer solutions with 12 wt.% PVA of all molecular weights blended with the colloidal dispersion in different compositions (20:80, 40:60, 50:50, 60:40, 70:30, 80:20) were electrospun and the obtained fiber morphologies are shown in Figure 7. It is evident that the composition of the spinning solutions had a significant impact on the morphology of the nanofibers. In all cases, improved morphological changes are observed with increasing amounts of PVA in the blend, resulting in an increase in the values of their rheological properties. However, at very high amounts of PVA (70:30 and 80:20) in the blends of HMW, the uniformity decreases. Therefore, we conclude that if the PVA fraction is less than 40% in the blend, beaded fibers are formed, while fiber uniformity improves from the ratio of 50:50 with the best uniformity being achieved at 60:40 of HMW.

### 2.7. In Vitro Release Studies

**Figure 8:**
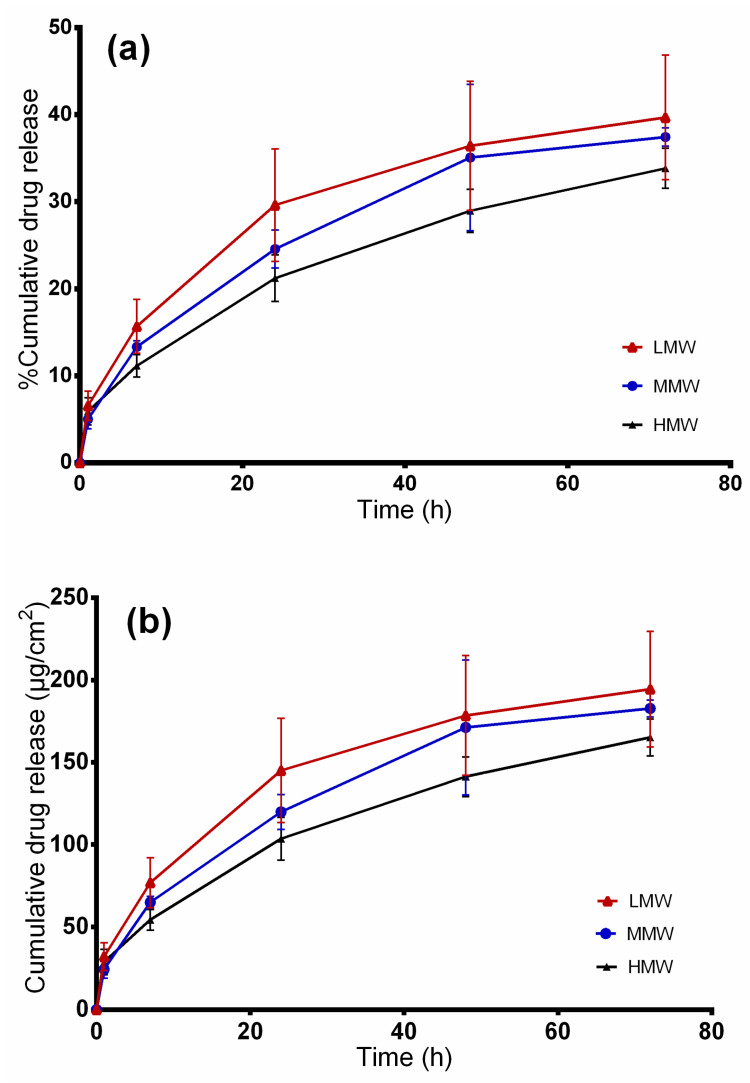
In vitro drug release studies. (**a**) Percentage of released betulin and (**b**) amount of betulin released from TE-loaded wound dressings.

Electrospun fiber mats prepared from blends with 12 wt.% PVA solutions of different molecular weights and colloidal TE dispersions (60:40) were subjected to release studies using modified Franz diffusion cells. Figure 8 depicts the cumulative amount of betulin released within 72 h. As can be seen, nanofibers from LMW showed higher and quicker release of betulin (194.46 ± 35.1 µg/cm^2^), followed by MMW (182.67 ± 5.2 µg/cm^2^) and HMW (165.13 ± 11.2 µg/cm^2^) electrospun fibers. This effect was attributed to the fiber morphologies and diameters. Blends from both molecular weights, LMW and MMW, exhibited a mat of thinner fibers making it easier for the drug to diffuse through. Between LMW and MMW, no significant differences were observed. On the other hand, the blend of HMW showed thicker fibers and drug molecules have to travel a longer distance through the polymeric matrix leading to a slower release [50]. Release profiles between LMW and HMW differed significantly (*p* < 0.05) on the release of betulin from electrospun fibers.

Table 4 shows the modeling results obtained from the four kinetic models. On comparing the R^2^ values of the formulations, it was found that betulin release from the electrospun PVA could be best fitted by the Korsmeyer–Peppas model as this model showed higher R^2^ values than all the other models. From the Korsmeyer–Peppas model, n values were found to be between 0.41 and 0.48 for all dressings, indicating that betulin release from PVA/TE fiber mats can be largely described as matrix diffusion [51,52]. This is in perfect agreement with the spatial distribution of the colloidal TE dispersion within PVA found by confocal Raman micro-spectroscopy, indicating that a hydrocolloid matrix is formed [32]. A fiber mat can be assumed to be a multiparticulate matrix system where the overall TE release is largely affected by the size of the individual fibers which form a diffusion barrier [50,53].

### 2.8. Ex vivo Permeation through Wounded Skin

**Figure 9:**
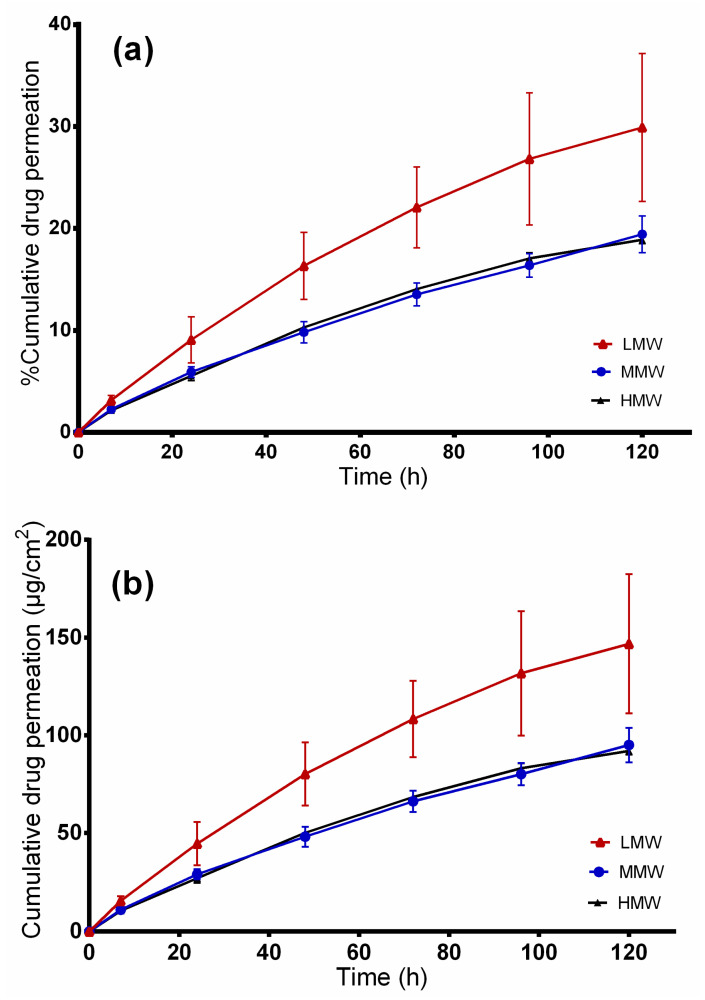
Ex vivo drug permeation studies. (**a**) Percentage of permeated betulin and (**b**) amount of betulin permeated from TE-loaded wound dressings.

The developed wound dressings are intended to be applied on wounded skin. Figure 9 shows the cumulative permeated amount of betulin from PVA/TE blend electrospun fiber mats in different molecular weights after 120 h. The cumulative amount of drug permeated from LMW was 146.81 ± 35.6, 95 ± 8.8 for MMW and 92 ± 1.8 µg/cm^2^ from the HMW/TE blend at 120 h. Again here, a higher and faster cumulative amount of drug permeated from the LMW formulation (permeation coefficient: 8.3 × 10^−2^ mg/cm^2^.h, *p* < 0.05), whereas no substantial differences were observed between MMW (permeation coefficient: 4.8 × 10^−2^ mg/cm^2^.h) and HMW (permeation coefficient: 5.4 × 10^−2^ mg/cm^2^.h) nanofibers. Electrospun fibers from MMW (241 nm) and HMW (392 nm), being thicker in diameter, demonstrated lower drug permeation in comparison to LMW (143 nm) where the fibers are thinner. 

Analysis of the permeation data showed that the kinetic can be fitted best to the Korsmeyer–Peppas model (R^2^: 0.9938 for LMW, 0.9997 for MMW and 0.9912 for HMW). From the Korsmeyer–Peppas model, n values were found to be between 0.74 and 0.77 for all the dressings, showing that betulin permeation from PVA/TE fiber mats is largely influenced by the diffusion through the skin even though the barrier had been removed. 

Drug release from the electrospun fibers and transport through the synthetic membrane is markedly faster (*p* < 0.05) in comparison to diffusion through the dermatomed skin. For instance, only 22% (108 ± 19.5 µg/cm^2^) of betulin from LMW/TE fibers permeated through the skin, whereas about 40% (194.46 ± 35 µg/cm^2^) was released through the synthetic membrane after 72 h. However, an influence of the kind of electrospun fibers is still visible. To put this in the context of wound healing, a controlled release from the PVA matrix is achievable in the very early phase of the treatment, whereas with increasing closure of the wound, the skin itself contributes to a sustained release stopping the permeation almost completely when the barrier function of the skin is restored [9].

## 3. Materials and Methods 

### 3.1. Materials

PVA with molecular weights of 67 (low molecular weight, (LMW)), 130 (medium molecular weight (MMW)) and 146–186 kDa (high molecular weight, (HMW)) (87–89% hydrolyzed) was purchased from Sigma Aldrich (Steinheim, Germany). All other materials were obtained from the named supplier. Hydrogenated phospholipids, Lipoid GmbH (PL90 H, Ludwigshafen, Germany), Sunflower oil, Caesar & Loretz GmbH (Hilden, Germany), Birch bark extract, (Amryt AG, Niefern-Öschelbronn, Germany), Parafilm^®^, Bemis Company Inc., (Oshkosh, WI, USA), Whatman Nuclepore polycarbonate membrane filters, Sigma-Aldrich, (Steinheim, Germany). Ultra-pure water (ELGA Labwater, Celle, Germany) was used as a solvent to prepare the aqueous solutions. Pig ears for ex vivo permeation studies were provided by the Department of Experimental Medicine, University of Tuebingen [54].

### 3.2. Preparing the Colloidal Dispersions

The colloidal dispersion with particle sizes of 400 ± 49 nm which was utilized in this work consisted of 2.5% PL90H, 1.0% SO and 0.5% TE as the dispersed phase and water as the continuous phase optimized from our previous study. Briefly, the dispersion was prepared using a two-stage homogenization process where a pre-dispersion is first formed using a rotor-stator system (Ultra Turrax T25, IKA, Staufen, Germany), at 9500 rpm with eventual homogenization using a high-pressure homogenizer (Emulsiflex C-3, Avestin, Mannheim, Germany) for 8 cycles at a pressure of 100 MPa [33]. 

### 3.3. Preparation of Solutions and Electrospinning of Nanofibers

From all molecular weights, PVA polymer solutions were prepared with varying concentrations (5, 7, 10, 12, 15 and 20 wt.%) by dissolving PVA in water at 90 °C under magnetic stirring. After PVA was completely dissolved, the solution was cooled to room temperature and used the following day. Subsequently, for electrospinning experiments, the colloidal TE dispersions were blended with the prepared PVA solutions in the ratio of 60:40 using a magnetic stirrer at 40 °C for 2 h to form a homogeneous solution. Besides, blends of colloidal dispersions/12 wt.% PVA solutions with different mass ratios (20:80, 40:60, 50:50, 60:40, 70:30 and 80:20) were prepared to test the influence of blend compositions on fiber morphology. Samples were then cooled down to room temperature prior to electrospinning. 

### 3.4. Electrospinning of Nanofibers

**Figure 10:**
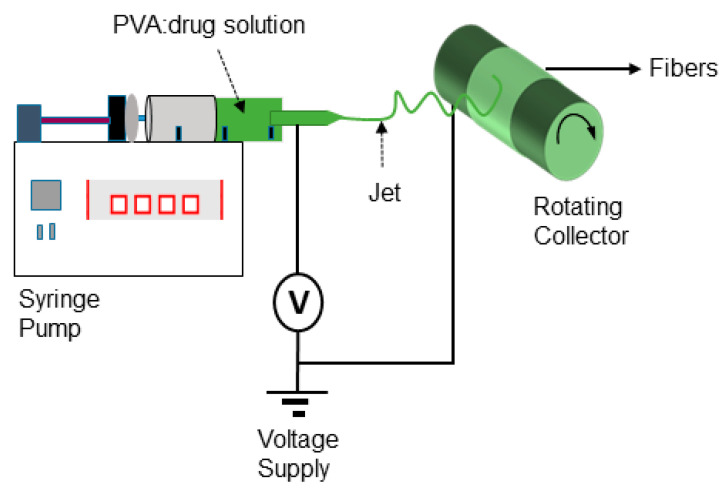
Schematic representation of the electrospinning setup used to fabricate nanofibers.

Electrospinning was performed using a conventional unit, Nanolab Instruments Sdn. Bhd. (Subang Jaya, Malaysia). Figure 10 shows a scheme of the electrospinning setup used to perform the experiments. A blunt-end needle (18-gauge) attached to a 5 mL plastic syringe was used, while the electrospinning conditions were a needle to collector distance = 10 cm, voltage = 15 kV and flow rate = 0.5 mL/h. A drum rotating collector covered with non-sticky aluminium foil and a speed fixed at 1000 rpm was used to collect the fibers. All electrospinning studies were carried out at ambient temperature (23 ± 2 °C) and a relative humidity of 45%.

### 3.5. Rheological Characterization of the Spinning Solutions

Rheological properties in the bulk were determined for all polymer solutions using a Physica MCR 501 rheometer (Anton Paar, Graz, Austria) with the cone-plate measuring system CP25-1 at a constant temperature of 25 °C. Rotational tests were performed in order to determine the viscosity of the solutions at a shear rate of 100 s^−1^.The amplitude sweep tests were performed to estimate the viscoelastic properties expressed as storage modulus G′ and loss modulus G″. The frequency was set at 1 Hz and the deformation was in the range of 0.01 to 1000%. Both the storage modulus G′ and loss modulus G″ were determined in the linear viscoelastic range (LVE).

### 3.6. Surface Tension and Conductivity

Surface tension was measured at room temperature by the Du Noüy ring method with a TD1C LAUDA tensiometer (Lauda-Königshofen, Germany). For each solution, average values of parameters from at least three measurements are reported. Conductivity of solutions was also measured at 25 °C using a WTW Conductivity meter 340i (Weilheim, Germany).

### 3.7. Qualitative Analysis of Electrospinning Process and Nanofiber Formation

A qualitative evaluation was done on the performance of polymer solutions during electrospinning using a plugged digital camera (Digimicro Profi, DNT, Dietzenbach, Germany). The camera was attached to the electrospinning process and so it was possible to visualize what was happening at the tip of the needle, like the formation of jet or droplets and Taylor cone behavior.

### 3.8. Characterization of Nanofibrous Scaffolds

The fiber morphology of both the blank and the TE-loaded fibers was observed by SEM, using a Zeiss DSM 940 A, Carl Zeiss GmbH (Oberkochen, Germany). Prior to SEM observation, each of the fiber mats (0.5 × 0.5 cm) was placed on a conductive double-sided tape and sputtered with gold using Biorad E 5100 Sputter Coater (Bio-Rad GmbH, Munich, Germany) at 2.1 kV and 20 mA for 240 s. Based on these SEM images, the average fiber diameters were determined from at least 30 measurements using ImageJ software (National Institute of Health, Bethesda, MD, USA). 

### 3.9. Ex Vivo Skin Permeation and In Vitro Drug Diffusion Studies

Modified vertical Franz diffusion cells with a reservoir capacity of 12 mL (Gauer Glas, Püttlingen, Germany) were used to conduct ex vivo permeation and in vitro release studies. In the case of in vitro release studies, synthetic polycarbonate membranes (pore size diameter of 0.4 µm) were used. For ex vivo permeation studies, pig ears were received from the Department of Experimental Medicine, University Hospital of Tuebingen right after the death of the animals. *The Department of Pharmaceutical Technology is registered for the use of animal products at the District Office of Tuebingen (registration number: DE 08 416 1052 21).* In ex vivo permeation studies, the skin was prepared according to previous studies [55,56]. Briefly, fresh pig ears were washed with isotonic saline using cotton balls and after postauricular skin excision, wrapped in aluminium foil and stored at −30 °C until further use. On the day of experiment, the skin samples were thawed at room temperature, cut into skin strips of 3 cm width and pinned to a block of Styrofoam pre-covered with aluminium foil. Thereafter, “wounded” skin samples were prepared by a skin grafting method using a Dermatom (Dermatom GA 630, Aesculap AG & Co. KG, Tuttlingen, Germany). Here, the skin was “wounded” through initial removal of the 0.2 mm outermost layers of the skin using Dermatom. Subsequently, the remaining skin was dermatomed to a thickness of 0.4 mm, and punched to obtain discs of 25 mm in diameter using a circular hole punch (Eduard Gottfried Ferne, Remscheid, Germany) [9]. 

Afterwards, the Franz diffusion cells equipped with synthetic membrane/porcine skin were filled with a mixture of ethanol and water, 50:50 (*v*/*v*), as the receptor medium. The diffusion area was 1.77 cm^2^. In all molecular weights, the final composition (wt.%) of PVA/colloidal dispersions (60:40) blends consisted of 7.2 wt.% PVA, 1 wt.% PL90H, 0.4 wt.% SO and 0.2 wt.% TE. In both experiments, samples of 30.50 mg fiber mats, exactly weighed, were loaded in the donor compartments and covered with parafilm to avoid solvent evaporation. The experiments were performed at 32 °C with continuous stirring at 500× *g*.

The amount of betulin diffused through the membrane/skin was determined by withdrawing samples of 1 mL from the receptor chamber at a predetermined time interval and replacing them with an equal volume of fresh prewarmed receptor medium to keep the sink condition. The samples were analyzed directly for betulin content using high-performance liquid chromatography with ultraviolet detection (HPLC-UV). The experiments were conducted in triplicate.

### 3.10. Betulin Permeation/Release Kinetics Assessment

The release/permeation kinetic of betulin from TE/PVA scaffolds was fitted by zero-order (Equation (1)), first-order (Equation (2)), Korsmeyer–Peppas (Equation (3)) and Higuchi (Equation (4)) [57]. After evaluating the regression coefficient (R^2^), the best mathematical kinetic model was obtained. In all cases, M_t_ is the amount of betulin determined in the receptor medium at the fixed times, and t is the time intervals.

Zero−order model, where K_0_ is the zero-order release constant:

M_t_ = K_0_t (1)

First-order model, where K_1_ is the first-order release rate constant:

In (1 − M_t_) = −K_1_t (2)

Korsmeyer−Peppas model:

Mt = Kt^n^ (3)

where K is the Korsmeyer–Peppas constant which is related to the characteristics of the delivery system and the encapsulated drug. The n is the diffusional exponent that shows the betulin release mechanism, where n = 0.45, a Fickian diffusion mechanism; 0.45 < n < 1, a non-Fickian diffusion mechanism (anomalous transport), in which both Fickian diffusion and Case-II transport occurs (Equation (3)) [58]. 

Higuchi model, where K is the Higuchi constant:

Mt = Kt^0.5^ (4)

### 3.11. HPLC Analysis

The LC-20A prominence HPLC system (Shimadzu, Kyoto, Japan) was used for betulin analysis. The mobile phase was composed of acetonitrile and water with the addition of 0.1% (*v*/*v*) phosphoric acid. For efficient betulin quantification, gradient conditions were used according to the isocratic/gradient modes developed by Armbruster et al. [59]. The flow rate was set to 1.2 mL/min, the injection volume was 100 µL and the oven temperature was 40 °C. For HPLC separation, a Nucleosil 100-5 C18 EC 125/4 column together with a precolumn Universal RP EC 4/3 (Macherey-Nagel, Düren, Germany) was used. The retention time of betulin was approximately 7.5 min and the detection wavelength was set at 210 nm.

### 3.12. Statistical Analysis

The obtained data were acquired from repeated experiments, at least three times, and statistically evaluated in terms of the mean and its standard deviation (mean ± SD). Statistical differences were determined by one-way or two-way analysis of variance (ANOVA) using GraphPad Prism 6.0 (GraphPad Software Inc., La Jolla, CA, USA). Results were considered to be statistically significant at the level of *p*-value less than 0.05.

## 4. Conclusions

This study broadens the knowledge on PVA-based electrospun fibers with a colloidal dispersion of a birch bark extract as the active principle for enhanced wound healing. The concentration and molecular weight of the polymer as the most decisive parameters have a significant effect on the rheological properties of the polymer solution and affect directly the resulting fiber properties. Overall, the viscosity of the prepared polymer solutions increased as the molecular weight and solution concentration increased. Incorporation of colloidal TE dispersions resulted in electrospun fibers of thinner diameters. The average fiber diameters in the whole studied design space varied between 143 and 2204 nm. It was also possible to control the release rate of betulin by just adjusting mainly the thickness of the electrospun fibers which can be adjusted by either, or both, the polymer concentration and molecular weight. TE was released much faster from fibers of LMW, while MMW and HMW performed better in terms of electrospinnability with more uniform fiber morphologies. We conclude that such developed scaffolds together with the positive benefits of birch bark extract present excellent dressing materials which can be tailored to the specific needs of enhanced healing of various types of wounds.

## Figures and Tables

**Table 1:** Physical-chemical characteristics of the dry birch bark extract used [9].

TE Composition	Specific Surface Area	Particle Size D50%
Betulin 81.60%, lupeol 2.08%, betulinic acid 3.84%, erythrodiol 1.05%, oleanolic acid 0.97%, Betulinic acid methyl ester 0.52%, undisclosed substances 9.94%	42 ± 0.4 m^2^/g	5.8 µm

**Table 2:** Fiber diameters of the obtained electrospun fibers from pure PVA solutions.

Molecular Weight	PVA (wt.%)	Average Diameter (nm) ± SD	Minimum Diameter (nm)	Maximum Diameter (nm)
**LMW**	5%	-	-	-
	7%	-	-	-
	10%	147 ± 24	91	202
	12%	174 ± 25	113	228
	15%	224 ± 22	178	271
	20%	390 ± 36	304	466
**MMW**	5%	183 ± 46	108	307
	7%	224 ± 35	162	276
	10%	372 ± 26	288	418
	12%	543 ± 70	437	670
	15%	655 ± 46	589	759
	20%	1357± 450	742	2333
**HMW**	5%	269 ± 24	226	311
	7%	358 ± 38	305	443
	10%	688 ± 94	482	863
	12%	975 ± 170	768	1364
	15%	1135 ± 405	762	2337
	20%	2204 ± 151	1500	2397

**Table 3:** Diameters of the obtained electrospun fibers from PVA/colloidal dispersions blends (60:40).

Molecular Weight	PVA (wt.%)	Average Diameter (nm) ± SD	Minimum Diameter (nm)	Maximum Diameter (nm)
**LMW**	12%	143 ± 29	88	225
	15%	187 ± 31	152	272
	20%	241 ± 28	191	298
**MMW**	10%	174 ± 22	129	225
	12%	241 ± 32	178	317
	15%	308 ± 29	263	373
	20%	424 ± 33	355	497
**HMW**	10%	319 ± 56	267	503
	12%	392 ± 41	341	499
	15%	605 ± 55	509	689

**Table 4:** In vitro release kinetics data of the TE-loaded electrospun fiber mats fitted to mathematical models.

Sample	Zero-Order	First-Order	Higuchi	Korsmeyer–Peppas Model	
	R^2^	R^2^	R^2^	R^2^	n
**LMW**	0.8674	0.8955	0.9518	0.9727	0.41
**MMW**	0.9062	0.9233	0.9705	0.9875	0.46
**HMW**	0.9504	0.9670	0.9947	0.9967	0.48
